# Epithelial-myoepithelial carcinoma of the hypopharynx: A rare case

**DOI:** 10.3892/ol.2014.2024

**Published:** 2014-04-02

**Authors:** MING GUAN, XIAOLIN CAO, WEI WANG, YONG LI

**Affiliations:** 1 Department of Otorhinolaryngology Head and Neck Surgery, Hangzhou First People’s Hospital, Hangzhou, Zhejiang 310006, P.R. China; 2 Department of Pathology, Hangzhou First People’s Hospital, Hangzhou, Zhejiang 310006, P.R. China

**Keywords:** epithelial myoepithelial carcinoma, hypopharynx

## Abstract

Epithelial-myoepithelial carcinoma (EMC) is a rare neoplasm, which predominantly arises in the parotid gland. EMC is characterized by two types of cells; myoepithelial and epithelial cells arranged in well-defined tubules. A 48-year-old male with a six-month history of dysphagia presented with a 2-cm-diameter mass in the left posterior wall of the hypopharynx. Histopathological examination revealed that the tumor cells were arranged in duct-like structures with an inner layer of ductal cells and an outer layer of clear cells. Immunohistochemically, the outer layer of clear cells stained positive for calponin, p63 protein, glial fibrillary acidic protein, S-100 protein and smooth muscle actin, which is consistent with a myoepithelial phenotype. The inner layer stained positive for cytokeratin and cytokeratin-7, which is consistent with an epithelial phenotype. The tumor was excised and no recurrence or metastasis was found 27 months following surgery. To the best of our knowledge, this is the first case of EMC described that has arisen from the hypopharynx.

## Introduction

Epithelial-myoepithelial carcinoma (EMC) is a rare neoplasm, first described in 1972 by Donath *et al* (1). EMC accounts for ~1% of epithelial salivary gland tumors, with the majority of cases of EMC arising in the parotid gland (2). Histologically, EMC is characterized by two types of cells arranged in well-defined tubules; epithelial cells in the inner layer and myoepithelial cells in the outer layer. EMC is always found clinically, and is occasionally identified by the patient or doctor as it commonly presents as a painless and slow growing neoplasm. In addition, the majority of patients have no complaints at the early stage. EMC is recognized to be a low-grade malignant tumor with a low metastasis rate and the primary treatment of EMC is to surgical excision (3). However, EMC arising in the hypopharynx has not previously been reported. The current study presents the first case in a 48-year-old male who exhibited no other symptoms with the exception of dysphagia for six months. The 2-cm-diameter mass was predominantly located in the left posterior wall of the hypopharynx, and the mass was histologically and immunohistochemically confirmed as EMC. The current study describes the treatment and outcomes of EMC in the hypopharynx and may provide useful information for future studies. Patient provided written informed consent.

## Case report

### Case summary

A 48-year-old male was referred to the Digestive System Department due to a 6-month history of dysphagia in February, 2011. A video-fiber gastroscopy revealed a 2-cm-diameter mass in the left posterior wall of the hypopharynx. False vocal folds, vocal folds and piriform fossa were normal, as were the other portions of the upper aero-digestive tract. A biopsy showed a minor salivary glands tumor. Computed tomography (CT) of the neck revealed a contrast-enhanced mass in the hypopharynx. The patient underwent partial hypopharyngectomy with a left-side neck dissection under general anesthesia. The partial neck dissection revealed 20 lymph nodes that were negative for metastases. The present patient has shown no sign of recurrence or metastasis during the 27 months of follow-up.

### Pathological findings

**Figure 1:**
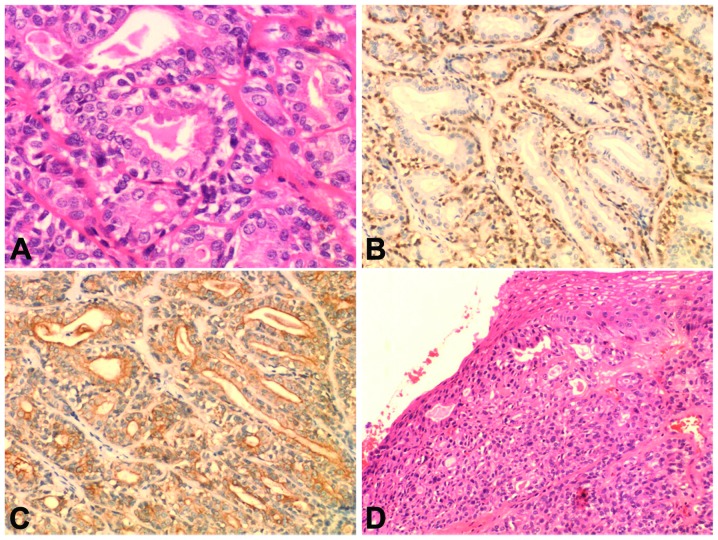
(A) Tumor characterized by well-defined tubules with two types of cells: An outer layer of myoepithelial cells with a clear cytoplasm and an inner lining of eosinophilic cuboidal epithelial cells (hematoxylin and eosin stain; magnification, ×200). (B) Outer myoepithelial cells showing immunoreactivity for S100 (magnification, ×100). (C) Inner cuboidal epithelial cells staining positive for cytokeratin (magnification, ×100). (D) The tumor cells are partially involved in the overlying pharyngeal epithelium, and induce necrosis and exfoliation (hematoxylin and eosin stain; magnification, ×100).

**Figure 2:**
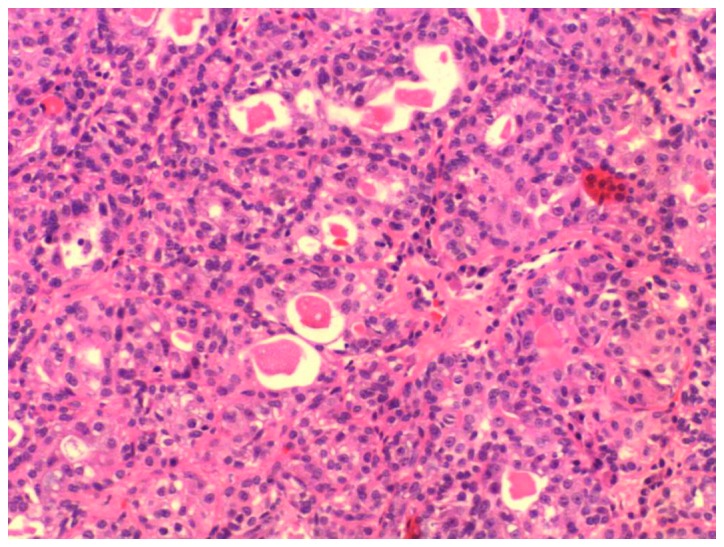
Tumor composed of variable sized ducts and nests. The tumor cells are monomorphic in appearance; round to oval (hematoxylin and eosin stain; magnification, ×100).

Microscopically, the tumor cells were arranged in duct-like structures with an inner layer of ductal cells and an outer layer of clear cells (Fig. 1A). Immunohistochemically, the outer layer of clear cells stained positive for calponin, p63 protein, glial fibrillary acidic protein, S-100 protein and smooth muscle actin, which is consistent with a myoepithelial phenotype (Fig. 1B). The inner layer stained positive for cytokeratin and cytokeratin-7, which is consistent with an epithelial phenotype (Fig. 1C). The tumor cells were partially involved in the overlying pharyngeal epithelium, and induced necrosis and exfoliation (Fig. 1D). These features were consistent with EMC. In the minor region, the tumor was composed of variable sized ducts and nests, and the tumor cells were monomorphic in appearance, round to oval and had a moderate amount of eosinophilic to clear cytoplasm (Fig. 2).

## Discussion

EMC is a rare neoplasm and the majority of cases occur in the parotid gland, while a few arise in the nasal cavity, paranasal sinus, nasopharynx, subglottic region, trachea, bronchus, lung, lacrimal gland, submandibular gland, tongue base, palate and liver (3). The majority of patients are females in their fifth to eighth decades (4). To the best of our knowledge, this is the first case of EMC occurring in the hypopharynx. The patient was male and had a 6-month history of dysphagia, without any pain or hoarseness.

The diagnosis of EMC is based on conventional light microscopy and immunohistochemical testing. Histologically, the EMC tumor is determined by well-characterized tubules of two cell types. These are an outer mantle of larger myoepithelial cells with a clear cytoplasm that stains positive for calponin, p63 protein, glial fibrillary acidic protein, S-100 protein and smooth muscle actin, that are surrounding an inner lining of eosinophilic cuboidal epithelial cells stained positive for cytokeratin, cytokeratin-7 and epithelial membrane antigen (3,5). By contrast, in the present case, certain areas of the specimens only consisted of variable sized ducts and nests, the tumor cells were monomorphic in appearance, round to oval and had a moderate amount of eosinophilic to clear cytoplasm, without the characteristic bilayered tubules, but similar to the characteristics of polymorphous low-grade adenocarcinoma. Initially it was believed that the diagnosis was a hybrid carcinoma composed of EMC and polymorphous low-grade adenocarcinoma. Therefore, this may cause diagnostic difficulty and confusion with other neoplasms if there were no characteristic bilayered tubules in the sampled specimens.

EMC is believed to be a low-grade malignancy tumor (6), and the first choice of treatment for EMC in the salivary glands is wide-surgical excision with a clear margin (7). Fonseca and Soares (3) reported that the recurrence rate of EMC is between 35 and 40%, and that the metastatic rate is between 8.1 and 25%. In the present study, the patient underwent partial hypopharyngectomy with a left-side neck dissection under general anesthesia. The partial neck dissection revealed 20 lymph nodes that were negative for metastases, and the patient has shown no sign of recurrence or metastasis during the 27 months of follow-up, however, close and prolonged follow-up is required to evalate the prognosis of EMC in the hypopharynx. A study by Cho *et al* (8) showed that the mean interval between diagnosis and recurrence was 5 years (range, 1–19 years) and that the mean interval between diagnosis and metastasis was 15 years (range, 4–20 years).

In conclusion, the present study reports the first case of an EMC in the hypopharynx. The patient has shown no sign of recurrence or metastasis during the 27 months since the partial hypopharyngectomy. The results of the current study may aid in the prognosis of EMC.
